# Well-scale demonstration of distributed pressure sensing using fiber-optic DAS and DTS

**DOI:** 10.1038/s41598-021-91916-7

**Published:** 2021-06-14

**Authors:** Gerald K. Ekechukwu, Jyotsna Sharma

**Affiliations:** Department of Petroleum Engineering, Patrick F. Taylor Hall, LSU, Baton Rouge, LA 70803 USA

**Keywords:** Applied optics, Optical techniques, Engineering, Fossil fuels

## Abstract

In this study, we used data from optical fiber-based Distributed Acoustic Sensor (DAS) and Distributed Temperature Sensor (DTS) to estimate pressure along the fiber. A machine learning workflow was developed and demonstrated using experimental datasets from gas–water flow tests conducted in a 5163-ft deep well instrumented with DAS, DTS, and four downhole pressure gauges. The workflow is successfully demonstrated on two experimental datasets, corresponding to different gas injection volumes, backpressure, injection methods, and water circulation rates. The workflow utilizes the random forest algorithm and involves a two-step process for distributed pressure prediction. In the first step, single-depth predictive modeling is performed to explore the underlying relationship between the DAS (in seven different frequency bands), DTS, and the gauge pressures at the four downhole locations. The single-depth analysis showed that the low-frequency components (< 2 Hz) of the DAS data, when combined with DTS, consistently demonstrate a superior capability in predicting pressure as compared to the higher frequency bands for both the datasets achieving an average coefficient of determination (or R^2^) of 0.96. This can be explained by the unique characteristic of low-frequency DAS which is sensitive to both the strain and temperature perturbations. In the second step, the DTS and the low-frequency DAS data from two gauge locations were used to predict pressures at different depths. The distributed pressure modeling achieved an average R^2^ of 0.95 and an average root mean squared error (RMSE) of 24 psi for the two datasets across the depths analyzed, demonstrating the distributed pressure measurement capability using the proposed workflow. A majority of the current DAS applications rely on the higher frequency components. This study presents a novel application of the low-frequency DAS combined with DTS for distributed pressure measurement.

## Introduction

Prediction of downhole pressures plays a vital role in a variety of applications including the management and evaluation of petroleum, geothermal, and groundwater resources. Traditionally, pressure is measured using gauges. While offering a cost-effective measurement solution, pressure gauges suffer from many limitations, such as frequent calibration needs, low tolerance in harsh environments (such as high-temperature, high-pressure, corrosive conditions, characteristic of petroleum and geothermal reservoirs^1^), hysteresis errors, and ability to only provide pressure at the discrete gauge location measuring at pre-determined points (in other words, single-point sensing).

Distributed fiber optics sensing (DFOS) is an advanced non-invasive, real-time sensing technology that can overcome many limitations of traditional gauges^2^. Among other things, fiber optic sensors are insensitive to electromagnetic noise, resistant to corrosion and high-pressure and high-temperature conditions, and do not require electronics along the optical path, making them suitable for many downhole sensing applications. The optical fiber functions both as the sensor and the channel to transmit the data, providing a truly distributed measurement in real-time along the cable. These sensors are capable of measuring physical properties such as temperature (via Distributed Temperature Sensing or DTS), vibration (via Distributed Acoustic Sensing or DAS), and strain (via Distributed Strain Sensing or DSS), simultaneously along the entire fiber.

Although DAS and DTS have been used for a variety of applications, ranging from flow profiling^2–5^, fracture monitoring^6–10^, seismic measurement^11–14^, leak detection^15,16^ and others, the use of a combination of DAS and DTS has not been explored for the prediction of downhole pressures. In this study, we develop and demonstrate a machine learning and signal processing-assisted workflow for continuous real-time distributed measurement of pressure using DAS and DTS data.

### DFOS overview and low-frequency DAS

**Figure 1:**
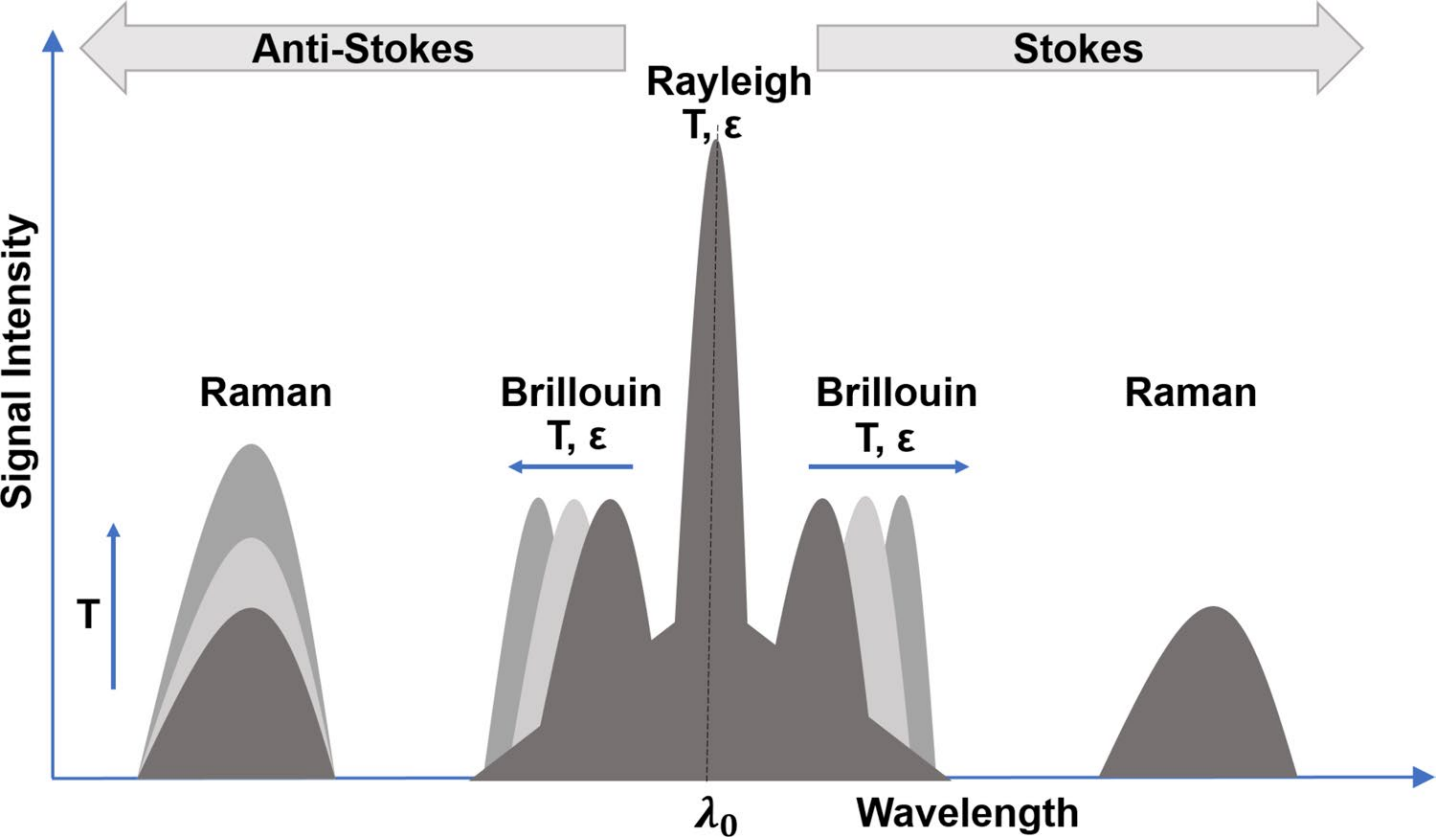
The backscattered light spectrum generated from typical DFOS.

DFOS utilizes the optical time-domain reflectometry (OTDR) principle to measure the spatial distribution of an intended measurand^17^. In the OTDR method, successive short laser pulses are sent out of the transmitter into the fiber, causing interactions with the heterogeneous crystalline core of the fiber optic cable which acts as scattering sites sending the backscattered light or signals back to the interrogation unit located at the surface. These impurities or molecular level heterogeneities are inherently and unintentionally produced during the optical fiber manufacturing. The interactions between the light and the scattering sites along the core of the fiber sense the temperature, vibration, and strain variation originating from events in the vicinity of the fiber-optic cable. The spectrum of the backscattered light consists of the Rayleigh, Brillouin, and Raman components (Fig. 1). Typically, the DTS uses the backscattered Raman component to measure temperature, DAS uses the backscattered Raleigh component to measure vibrations or dynamic strain, while DSS uses the Brillouin component to measure strain^18–20^.

Acoustic disturbance on a fiber generates microscopic elongation or compression of the fiber (micro-strain), which causes a change in the phase relation and/or amplitude. The raw DAS data are usually delivered in the form of delays in the optical phase [− π to + π] between two points along the fiber cable. The phase delay varies linearly with a small length change (the axial strain) between two locations separated by the gauge length. Differences in the strain obtained during successive pulses or the time differential of the measured optical phase (i.e., strain rate) may also be provided as a signal response by some sensing unit providers. ^21^.

A majority of the DAS applications discussed in the previous section have focused on the high-frequency bands (> 2 Hz) of the DAS data. The use of low-frequency DAS in the oil and gas industry is fairly recent and only a few researchers have explored it for practical applications. For instance, Jin and Roy^21^ used the low-frequency DAS data (< 0.05 Hz) to constrain the length, density, and width of reservoir fractures, while Becker et al. ^1^ employed a low-frequency DAS system to detect strain at mHz frequencies. Shragge et al.^22^ used low-frequency DAS for the acquisition of S-wave profiles. Technical improvements in optical interrogators have enabled improved SNR close to DC frequencies, so it is expected that the application space of low-frequency DAS will continue to grow. A unique characteristic of low-frequency DAS is that it is more sensitive to both the temperature and the strain effects, and thus the dynamic strain rates are clearly observed^23^. Thus, in principle, the low-frequency DAS phase data has a more direct relationship with pressure, which is further discussed in the next section and also demonstrated in the workflow presented in this study.

### Theoretical background

When light travels through a fiber of length *L* and refractive index *n*, the optical phase $\mathrm{\varnothing }$ is related to the wavenumber *k* by the following expression:

1 $$\phi = nkL,\,{\text{where }}\,\,k = 2\pi /\lambda$$

Direct pressure exposure induces changes in a phase differential $d\mathrm{\varnothing }/\mathrm{\varnothing }$ which changes the properties of the optical fiber. The changes in the optical phase induce strain ($dL/L or {\varepsilon }_{zg})$, modifies the index of refraction ($dn/n$) of the material (the photo-elasticity effect), and causes waveguide dispersion $(dk/k)$ as shown below^23,24^:

2 $$d\mathrm{\varnothing }/\mathrm{\varnothing }= dL/L+dn/n+dk/k$$

Hocker^24^ showed that the third term representing waveguide mode dispersion effects is negligible. The phase delay induces strain as shown below with $dn/n$ now represented by the second term of the left side of the equation below ^25,26^.

3 $$\frac{d\phi }{\phi }={\varepsilon }_{zg}-\frac{{n}^{2}}{2}\left[\left({P}_{11}+{P}_{12}\right){\varepsilon }_{rg}+{P}_{12 }{\varepsilon }_{zg}\right]$$

where *P*_*ij*_ is the strain optic (elastooptic or Pockels) coefficients and ${\varepsilon }_{zg}$ and ${\varepsilon }_{rg}$ are the axial and the radial components of the induced strain in the fiber, respectively. Accounting for double transit, substituting Eq. 1 into Eq. 3 and rearranging the equation, the strain sensitivity is given as^27^:

4 $${\varepsilon }_{zg}=\lambda d\phi /2\pi nL\xi$$

where,

5 $$\xi =1-\frac{{n}^{2}}{2}\left[{P}_{12 }-{v}_{g}\left({P}_{11}+{P}_{12}\right)\right]$$

Equation (4) above shows the relationship between the pressure-induced strain and the phase differential. Budiansky^23^ then further provided an expression for pressure sensitivity due to the induced strain as follows:

6 $${\varepsilon }_{zg}/p=-\frac{1-2\left(1-f\right){v}_{p}-2f{v}_{g}}{f{E}_{g}+(1-f){E}_{p}}$$

The expression has been deemed accurate for $f={\left(a/b\right)}^{2}\ll 1$ and ${E}_{p}\ll {E}_{g}$. Where, *a* is the radius of the fiber, *b* is the radius of the coating, ${E}_{g}$ and ${v}_{g}$ are the Young’s modulus and the Poisson ratio of the glass, respectively, while ${E}_{p}$ and ${v}_{p}$ are the Young’s modulus and the Poisson ratio of the cladding, respectively. Hughes and Jarzynski^28^ showed that the sensitivity is generally governed by both the bulk and the Young’s moduli of the coating materials, which are also temperature dependent. Furthermore, Giallorenzi et al^26^ observed that the pressure sensitivity of coated fibers can be frequency-dependent and this dependency is affected by the combined or synergistic effects of all the coatings and the fiber cable.

DAS measurements based on Rayleigh backscattering are temperature and strain dependent. However, the way it affects the measurement is different for both strain and temperature. The strain affects the measurement by directly changing the actual fiber length, but also through changes of the refractive index (photo-elastic effect). The temperature affects the measurement, again through changes of the fiber length (thermal expansion), but also through changes of the refractive index (thermo-optic effect). So at all times, the DAS measurement is affected by both strain and temperature. However thermal changes typically have a response time much slower than strain changes, and hence will have a much lower frequency content^18^. Jin and Roy^29^ also observed that the low-frequency component of DAS signals are affected by thermal perturbations and proposed the following relationship of low-frequency (LF) DAS with temperature and strain variations:

7 $$LFDAS={C}_{1}\Delta \varepsilon +{C}_{2}\Delta T$$

where $\Delta \varepsilon$ and $\Delta T$ are the strain and the temperature variations, respectively. ${C}_{1}$ and ${C}_{2}$ are coefficients dependent on the fiber structure and material properties^30^. ${C}_{2}$ can be significantly dependent on the thermal expansion coefficients of the entire multilayer structure of the fiber which could vary from fiber to fiber.

The above discussion establishes the physical dependencies between the pressure perturbations and the DAS-based measurements. However, the above equations also highlight the complex and often non-linear relations that depend on the fiber and the coating material properties (such as, thickness, elasticity, strain-optic coefficients etc.) and the dynamic environmental conditions (such as temperature and frequency effects), which may not be fully known analytically without assumptions or limitations. Machine learning algorithms have been demonstrated to effectively “learn” the complex non-linear relationships between a given set of target prediction output and input features. Thus, a machine learning approach was adopted in this study to directly learn the relationship between pressure and the DAS and DTS measurements using the observed data.

## Data acquisition

### Experimental set-up

**Figure 2:**
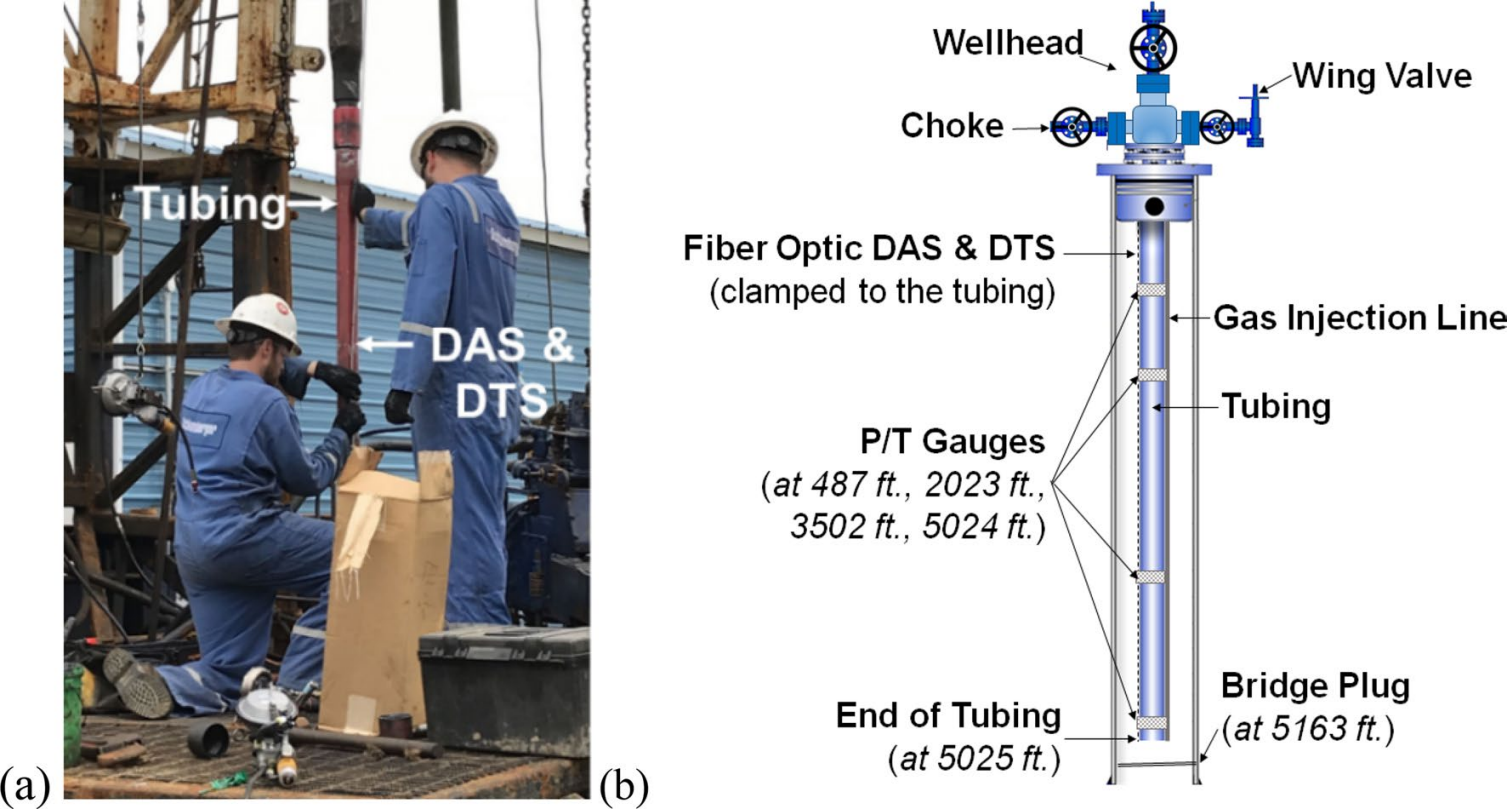
(**a**) DAS and DTS installation on the test-well at PERTT. (**b**) Test-well schematic showing the location of the four downhole gauges and fiber-optic DAS and DTS ^32^.

The data analyzed in this study was obtained from two-phase (nitrogen gas and water) flow experiments conducted in a 5163-ft deep test-well located in the Petroleum Engineering Research and Technology Transfer (PERTT) lab facility at LSU (Fig. 2a). The wellbore consists of a 9.625 in diameter casing that is cemented in place, with a 2.875 in diameter concentric tubing to 5025 ft. depth (Fig. 2b). DAS and DTS fiber cables, along with four downhole pressure and temperature (P/T) gauges are attached to the outside of the tubing as shown in Fig. 2b. The data acquisition parameters and sensor specifications are summarized in Table 1.

**Table 1:** Data acquisition and sensor parameters^32–34^.

DTS		DAS		Pressure and temperature gauge	
Optical Mode	Multi-mode	Optical Mode	Single-mode	Pressure Accuracy	< ± 1.0 psi
Spatial Resolution	3.28 ft	Spatial Resolution	4.92 ft	Pressure Resolution	0.035 psi
Sampling Interval	1.64 ft	Sampling Interval	2.53 ft	Temperature Accuracy	± 0.27 ^○^F
Temporal Resolution	12 s	Temporal Resolution	10 s	Temporal Resolution	1 s
Range	15 km	Range	16 km	Maximum Pressure	10,000 psi
Accuracy	± 1.8 °F	Frequency	10 kHz	Maximum Temperature	302 ^○^F

The DAS was acquired at a frequency of 10 kHz, hence, the maximum frequency we can measure is 5 kHz based on the Shannon-Nyquist criterion ^31^. To obtain the different frequency components of the signal, spectral decomposition is performed on the raw DAS time-domain data by applying the Fast Fourier Transforms. The frequencies are then split up into pre-specified bands consisting of different frequency ranges and then called the frequency band energy (FBE) data. It is preferred to analyze the DAS data in the FBE domain as it provides a simplified snapshot of the acoustic energy over a fixed duration and over different frequency ranges at any given time^23^. FBE data is also much smaller in size as compared to the original time-domain DAS data, making it easier to identify signals of importance and interpret vibration data only on those particular signals, leading to a significant reduction in turnaround time for data interpretation^23^. For this study, we would analyze seven different frequency bands as follows: Band-LF [0–2 Hz], Band-0 [2–5000 Hz], Band-1 [2–10 Hz], Band-2 [10–50 Hz], Band-3 [50–200 Hz], Band-4 [200–500 Hz], and Band-5 [500–1000 Hz]. The acoustic energy contained in frequency bands above 1000 Hz is insignificant in our specific dataset and they were therefore not analyzed^22^.

### Experimental procedure and datasets analyzed

Two-phase flow experiments using water and nitrogen gas were conducted in the test-well to understand gas–water flow dynamics at well-scale conditions. The wellbore was initially filled with water in both the tubing and the casing, and a fixed volume of nitrogen gas (measured in barrels or bbl) was injected either down the tubing or the 0.5 in diameter gas injection line strapped to the tubing (as shown in Fig. 2b). The objective of the experiments was to observe and characterize the gas rise in water using the fiber-optic sensors and downhole gauges as described in detail in the references^32,33^.

Two different experimental datasets were used in this study to demonstrate the proposed distributed pressure measurement workflow using DTS and DAS. The datasets correspond to different gas injection volumes, water circulation rates, backpressure, and injection method, as summarized in Table 2. The injection sequence is as follows: preconditioning stage—which involves the injection of water down through the tubing and up the annulus back to the surface with the objective of removing any leftover gas from the wellbore, gas injection stage—which is the injection of a fixed volume of nitrogen gas slug either down the injection line or down the tubing, and the post-injection or simply water circulation stage—in which water is once more injected to displace the gas in the well. The objective was to demonstrate that the methodology works for different operational conditions. In the first experiment (Dataset-1) nitrogen was injected through the gas line and allowed to rise to the surface through the annulus without any water circulation and with the choke closed at the surface, while in the second experiment (Dataset-2) the gas injection down the tubing is immediately followed by water injection to push the gas down the tubing and eventually up through the annulus and back to the surface, while a constant backpressure is maintained on the casing at the surface. Both two-phase flow scenarios will create some pressure disturbance that will be recorded by the pressure gauges. The main objective of this work is to model the relationship between the DAS and DTS values to the pressure gauge readings and then use the developed model to predict pressure at different depths along the wellbore for distributed pressure measurement. The temperature data read by the downhole gauges was only used for the DTS depth calibration.

**Table 2:** Experimental parameters used for the two datasets.

Dataset	Water circulation rate [GPM]	Gas injection volume [BBL]	Injection method	Backpressure [PSI]
1	0	2	Injection Line	Choke closed
2	100	5	Tubing	300

**Figure 3:**
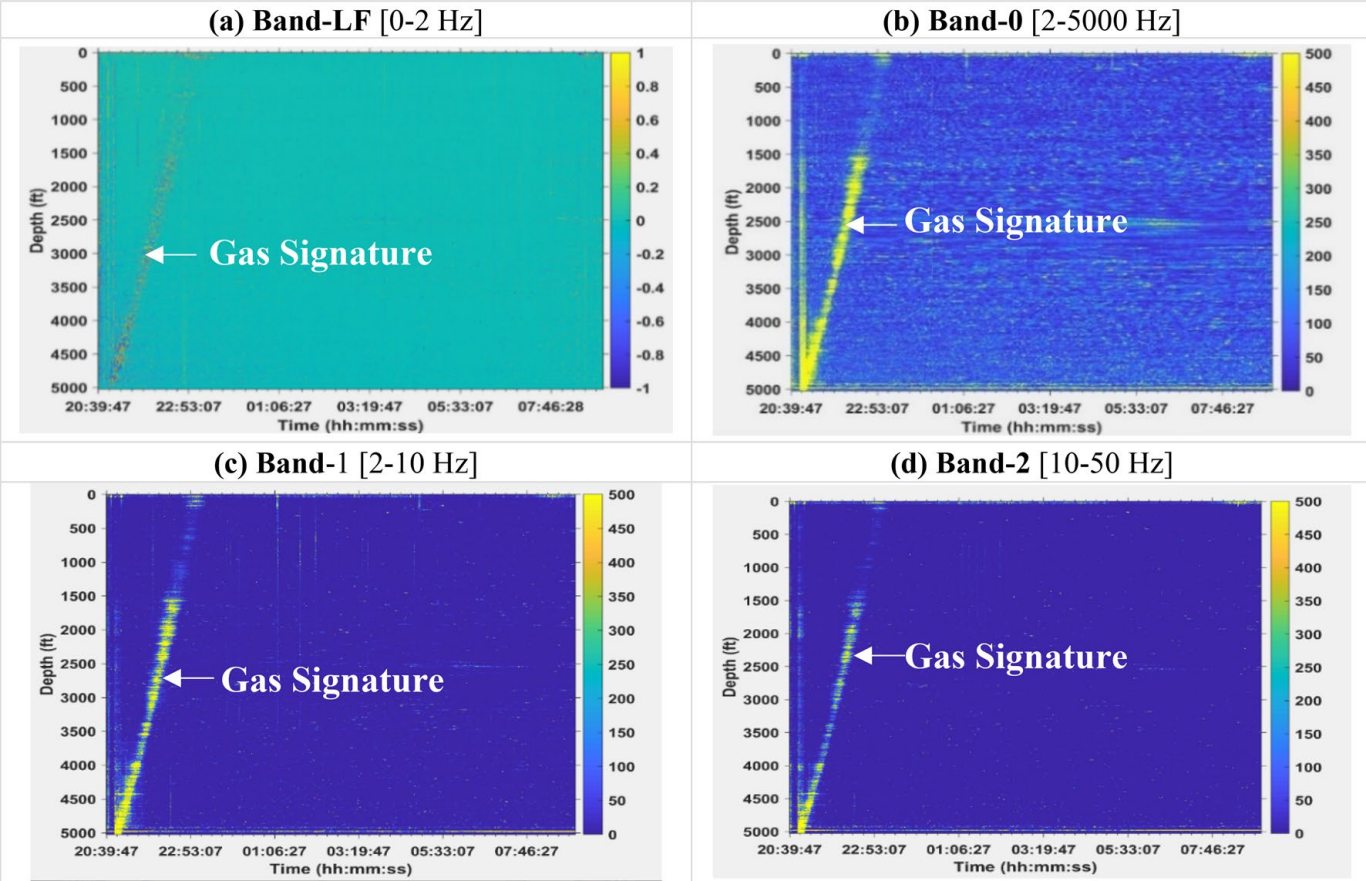
Waterfall plots showing the DAS values in the different frequency bands for Dataset-1.

**Figure 4:**
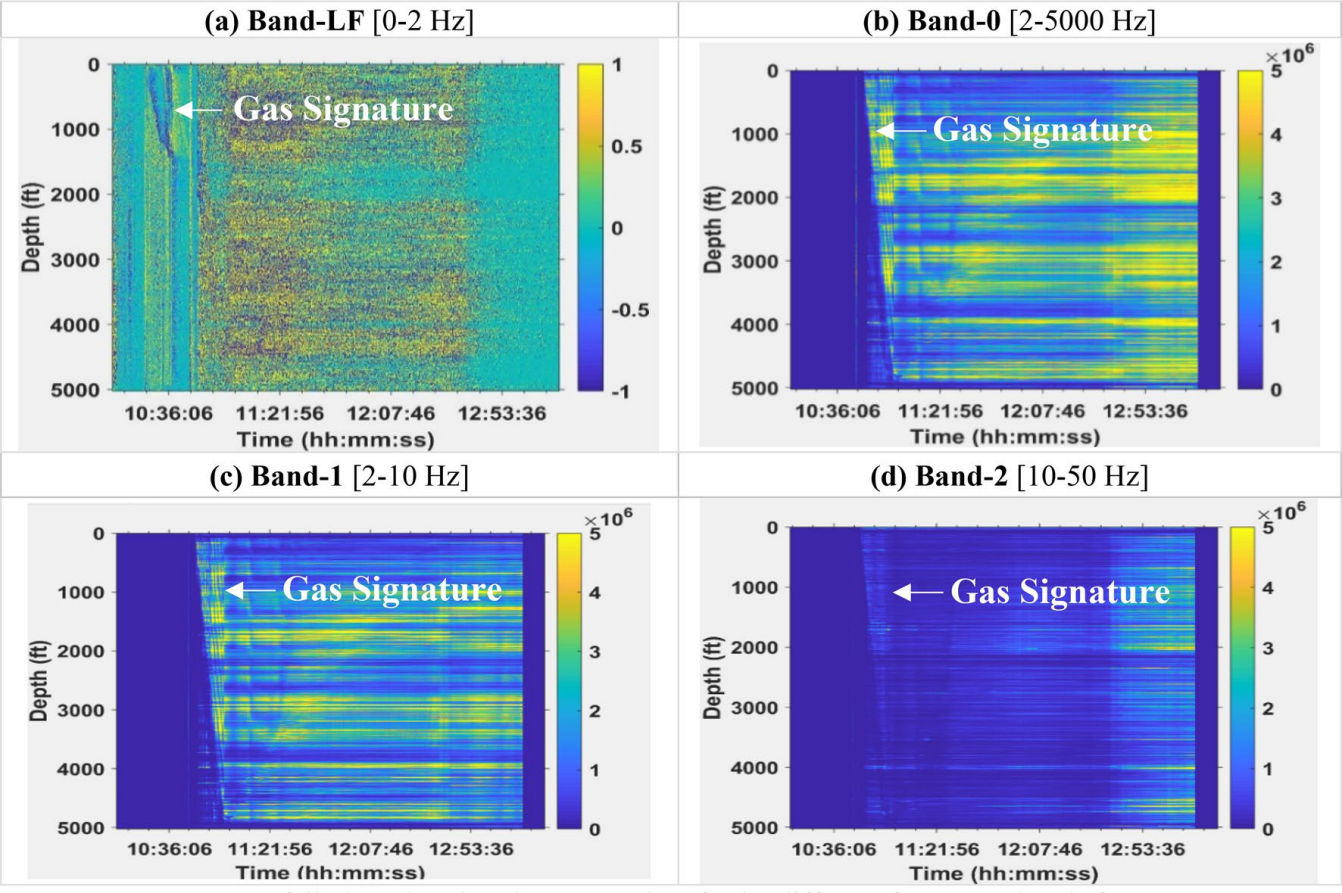
Waterfall plots showing the DAS values in the different frequency bands for Dataset-2.

Figures 3 and 4 show the DAS waterfall plots for Bands-LF, 0, 1, and 2 for Dataset-1 and Dataset-2, respectively which indicate the gas flow signature in water which is observed more clearly in Dataset-1 (with no water circulation) as compared to Dataset-2 (with water circulation). Plots for DAS Bands 3 to 5 are included in the supplementary material (Figs. S1 and S2). A detailed interpretation of the gas signature can be found in the references^33^. Since the LFDAS data are sensitive to both dynamic strain and temperature changes, Band-LF (0 to 2 Hz) has both positive and negative numbers depending on whether the fiber section is experiencing compressive or tensile strain or heating or cooling phenomenon^32^.

**Figure 5:**
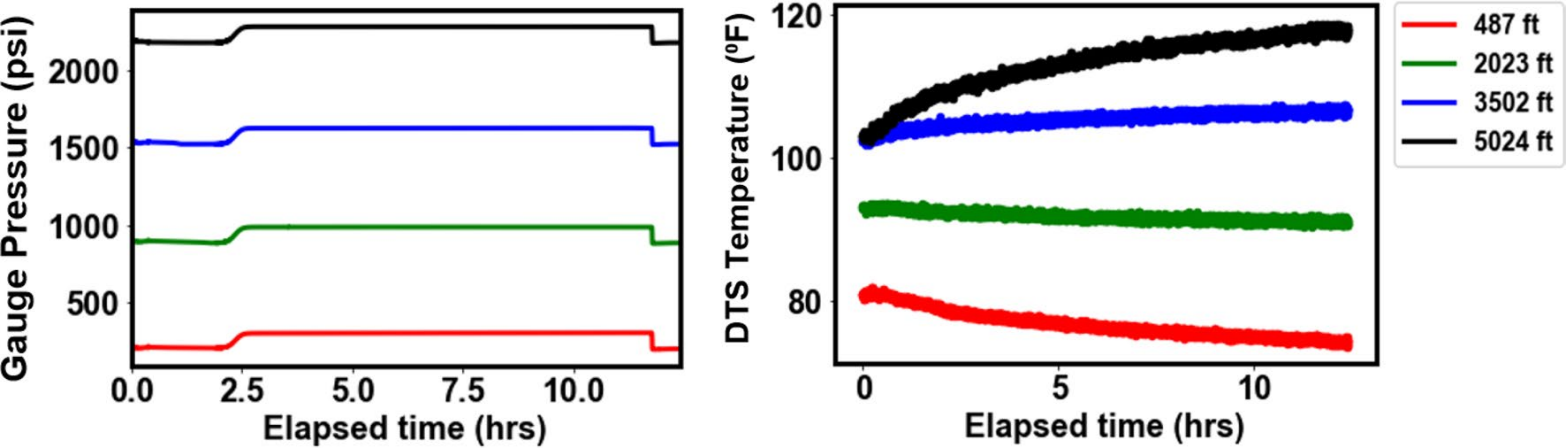
Pressures from downhole gauges and temperature from DTS at the four gauge locations for Dataset-1.

**Figure 6:**
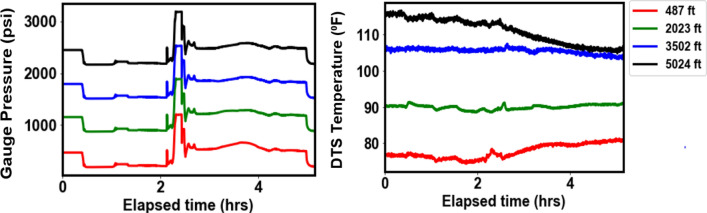
Pressures from downhole gauges and temperature from DTS at the four gauge locations for Dataset-2.

Figures 5 and 6 show the pressure and temperature profiles for Dataset-1 and Dataset-2, respectively, at the four depths where the gauges are installed (487 ft., 2023 ft., 3502 ft., and 5025 ft.). The pressure and temperature data in Dataset-1 spanned a period of about 12 h while that of Dataset-2 was about 5 h. For Dataset-1, the effect of gas was observed at about 2.5 h elapsed time, once the gas is in the annulus. The elapsed time is the time difference between any given time and the reference time, where the reference time corresponds to the start of the preconditioning stage described earlier. As expected, the pressures at the different gauges are lower for the top gauge and increases as we go deeper into the well. This is a result of hydrostatic pressure which increases as the depth increases. In addition, as the gas migrates out of the annulus to the surface we see that the pressure increases since a lighter fluid (nitrogen) is being replaced by a heavier one (water) in the annulus. The gas rise signature and arrival at surface are clearly observed in the DAS plots in Figs. 3 and 4. For Dataset-1, the decrease in pressure at about 11.5 h of elapsed time was a result of opening of the choke valve at the surface which marks the end of the experiment. For Dataset-2, which involves continuous water circulation post gas-slug injection, additional pressure effect arises due to turbulence flow, which are also evident in the high vibration energy in the DAS plots in Fig. 4. The maximum temperatures at the different gauges also showed an increasing trend down the wellbore as expected from the geothermal gradient. The temperature readings in Dataset-2 are a few degrees lower than Dataset-1 due to the cooling effect from the water circulation (at 100 GPM, see Table 2). In Dataset-2 the pressures were more erratic than those for Dataset-1 due to the dynamic flow effects resulting from the water circulation.

### Data preparation

One of the key steps in the data preparation was to align the downhole sensor data spatially and temporally. As summarized in Table 1, DAS, DTS, and the pressure gauges had sampling times of 10 s, 12 s, and 1 s, respectively. While the DTS and DAS produced distributed measurements every 1.64 ft and 2.53 ft, respectively, along the fiber, the pressure gauges measured pressure at only four discrete locations (487 ft., 2023 ft., 3502 ft., and 5025 ft.). The downhole temperature gauge data in our case was only used for depth calibration of the DTS. The first data preparation step was that the three different datasets had to be resampled to ensure that they had the same sampling interval and corresponding timestamps. Therefore, in order to prepare the data points to use in the machine learning model, the DAS and DTS were time-matched with a criterion that the DTS is matched with the DAS if their timestamps are within ± 3 s apart. This is a reasonable criterion since the temperature is not changing rapidly (Figs. 5 and 6). For some machine learning algorithms, the features or input variables in the dataset need to be transformed via normalization. Normalization ensures fast convergence of the gradient-based learning process, such as neural network models. Min–max scaling was performed on one feature at a time to scale the data (*y*_*i*_) to [-1, 1] using the following equation:

8 $${y}_{i}^{^{\prime}}=2\frac{{y}_{i}- {y}_{min}}{{y}_{max}- {y}_{min}}-1$$

The chosen machine learning method used in the main body of our work – the random forest—is robust and its accuracy remains the same with or without normalization.

## Methodology

### Analysis workflow

**Figure 7:**
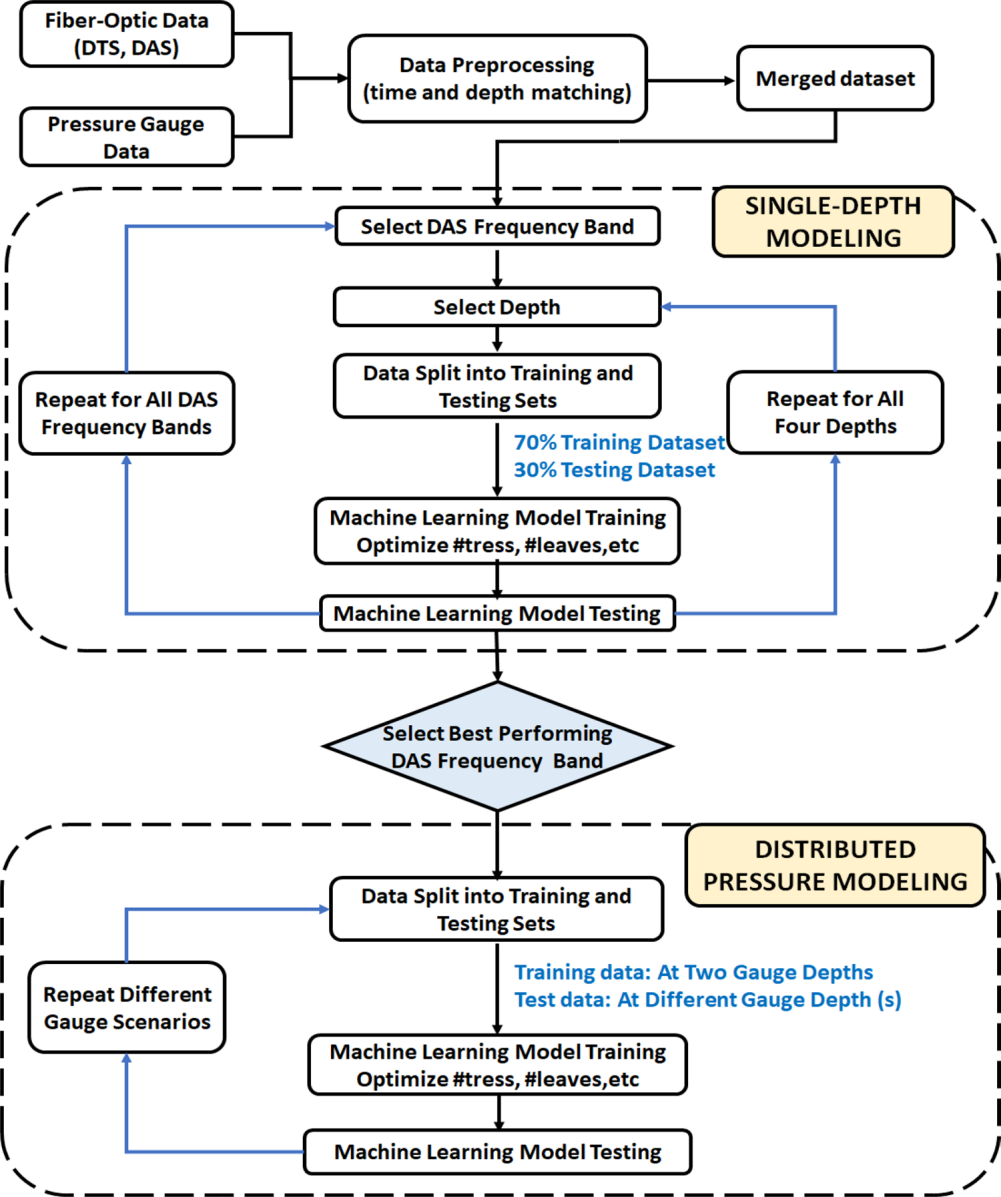
Workflow for pressure prediction using DTS and DAS.

The analysis workflow developed in this study is illustrated in Fig. 7 and described as follows:

(1) **Data preparation** DTS, DAS, and pressure gauge data is time and depth matched and normalized.

(2) **Single-depth analysis** The machine learning model is implemented independently at the four gauge depth (487 ft., 2023 ft., 3502 ft., and 5025 ft.). At each depth, the input features for the model are the DAS (one frequency band at a time) and DTS data, while the target output variable is the change in pressure relative to the initial pressure at the first time-step (∆P). 70% of the data were randomly selected for model training and the remaining 30% is used for blind testing. The performance is evaluated for each frequency band individually to select the one with the best performance for pressure prediction. This analysis is repeated at all four gauge depths and all seven frequency bands, for the two experimental datasets.

(3) **DAS frequency band selection** The best performing frequency band is selected based on the single-depth analysis at all four gauge locations, for both datasets. This frequency band is used for the distributed pressure analysis.

(4) **Distributed pressure analysis** Here the objective is to predict pressure at different depths using the DAS and DTS data. The machine learning model is trained using data at any two gauge depths and then blind-tested for predicting the pressures at the other gauge depths different from the ones used for training. The input features here are DTS, DAS (only the frequency band selected in (3), and elapsed time, and the target predicted is the change in pressure (∆P).

### Random forest algorithm

Five different machine learning algorithms were considered for our workflow including random forest^35,36^, gradient boosting machine (GBM)^37,38^, extreme gradient boosting (XGBoost)^39^, support vector regression (SVR)^40,41^, and different architectures of shallow artificial neural network (ANN). Of these, the random forest algorithm was selected as the model of choice in the main body of this work based on the consistently high performance (high R^2^ and low RMSE) and low computational time when compared with the other algorithms. The results of the comparison are presented in the supplementary material (Table S1 and Fig. S3).

**Figure 8:**
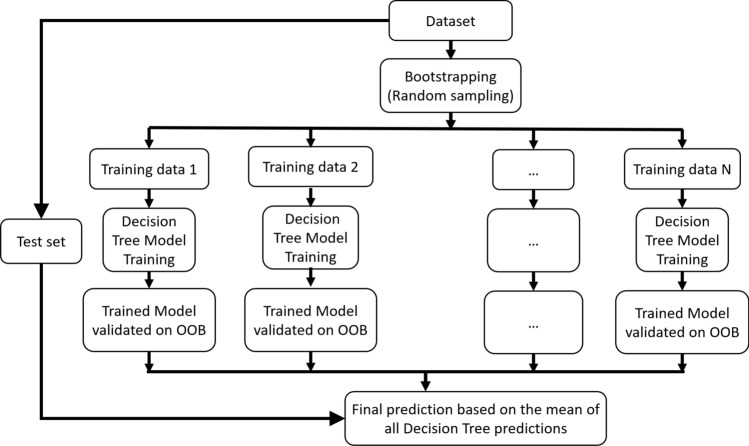
Schematic illustration of the random forest algorithm.

Random forest is an ensemble machine learning technique based on several decision trees ^35,36^ (Fig. 8). First, the dataset needs to be split into training and testing or evaluation datasets. The training set is then sampled randomly based on the number of decision trees to be trained. Each subset of the training set is further split into training and validation datasets (otherwise known as out-of-bag or OOB samples). Each decision tree builds its own model and uses the validation samples for evaluation. The decision tree model is a sequence of rules based on the features (nodes) and splitting criteria. All input variables and possible split points are evaluated and the split points that minimizes the cost function (mean squared error or MSE) across all training samples and validation samples are selected. The cost function is calculated as:

9 $$Cost function=\frac{1}{n} \sum_{i=1}^{n}{\left({y}_{i}-{\widehat{y}}_{i}\right)}^{2}$$

where ${y}_{i}$ and ${\widehat{y}}_{i}$ are the actual target and predicted target values, respectively, and *n* is the number of samples. Decision trees have several advantages in that they implicitly perform feature selection, they are not affected by the non-linearity of the predictors and they are relatively easy to interpret. However, they suffer from high variance, that is if we split the data set into two parts at random and then try to train on them, the results could be very different. Hence, in order to build a model with low variance and better accuracy, the ensemble approach is used to combine several decision tree models to obtain a stronger model. The ensemble methods usually involve creating multiple different subsets from the training data, building multiple predictive models, and then combining the predictions. The random forest employed in this work is based on the bootstrap aggregation or bagging for short^35^. Bagging involves bootstrapping the training data to get subsets, learn one model for each set which is usually run in parallel, and then average the model prediction.

The most important hyperparameters that need to be considered in the random forest modeling procedure are as follows:

- **Number of trees**: This is the number of trees that are used in the algorithm. The number of decision trees used in this study was 100 based on a parametric study that showed no appreciable improvement in the performance scores beyond this value.
- **Splitting criteria**: The mean square error or MSE was used as the splitting criteria.
- **Stopping criteria:** This can be specified by either the maximum depth of each tree or the minimum samples required to split an internal node. If the maximum depth is specified, then the splitting stops after the specified value is reached otherwise if the nodes are expanded until all leaves are pure or until all leaves contain less than minimum samples for a split. For this study, there was no performance improvement beyond a maximum depth of 10.
- **Minimum Sample Split:** The minimum samples required to split an internal node is 2 and the minimum number of samples to be in a leaf node is 1.

### Performance metrics

We employed the coefficient of determination or R-squared (R^2^) and the root mean squared errors (RMSE) to quantify the performance of our models. These performance metrics are robust enough to give the relative performance across the different scenarios and have been widely used in machine learning model performance assessment. They are calculated as:

10 $${R}^{2}=\frac{SSR}{SST}=\frac{{\sum }_{i=1}^{n}{\left({\widehat{y}}_{i}-\overline{y }\right)}^{2}}{{\sum }_{i=1}^{n}{\left({y}_{i}-\overline{y }\right)}^{2}} \, and\,RMSE= \sqrt{\frac{1}{n} \sum_{i=1}^{n}{\left({y}_{i}-{\widehat{y}}_{i}\right)}^{2}}$$

where ${\widehat{y}}_{i}$ is the predicted value, ${y}_{i}$ is the actual target value, $\overline{y }$ is the mean of the target values, and *n* is the number of samples. SSR is the "regression sum of squares" and quantifies how far the estimation is from the target feature mean prediction (based on no relationship with predictors). SST is the "total sum of squares" and quantifies how much the data point vary around their mean.

## Results

Table 3 summarizes the input and output features used for the machine learning models for the single-depth and distributed pressure modeling steps. We first present the results of the exploratory data analysis of the input and output variables to identify patterns and data distributions. This is followed by the discussion of results from the random forest models for the single-depth analysis at each gauge depth and the distributed pressure prediction scenarios.

**Table 3:** Machine learning model features for the single-depth and distributed pressure modeling.

Random Forest Model	Predictor (Input variables)		Target (Output variable)	Number of datapoints (Training/Testing data)
Single-depth analysis	DAS (all frequency bands), DTS		∆P	2668/1144 (Dataset-1)
				553/238 (Dataset-2)
Distributed depth analysis	DAS (Band-LF), DTS, Time		∆P	7624/7624 (Dataset-1)
				1582/1582 (Dataset-2)

### Descriptive data exploration

**Figure 9:**
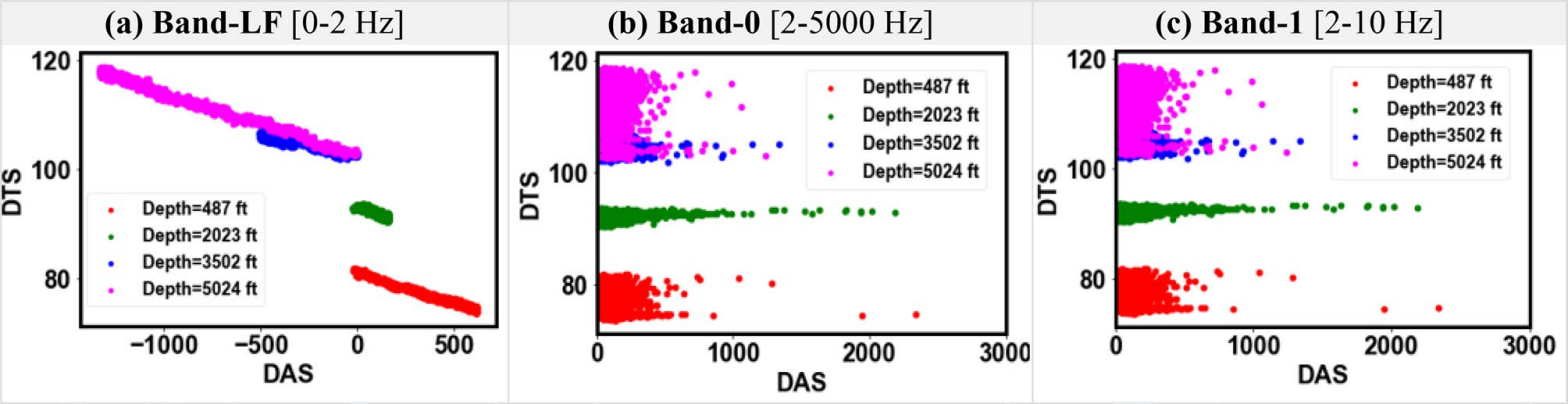
Cross-plots of input features DTS and different DAS frequency bands for Dataset-1.

**Figure 10:**
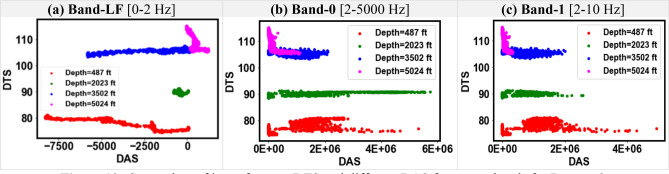
Cross-plots of input features DTS and different DAS frequency bands for Dataset-2.

**Figure 11:**
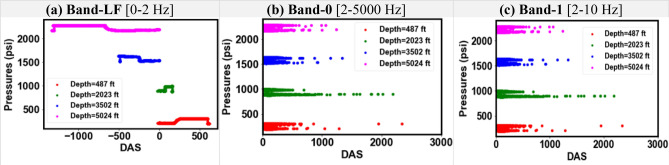
Cross-plots of pressure and different DAS frequency bands for Dataset-1.

**Figure 12:**
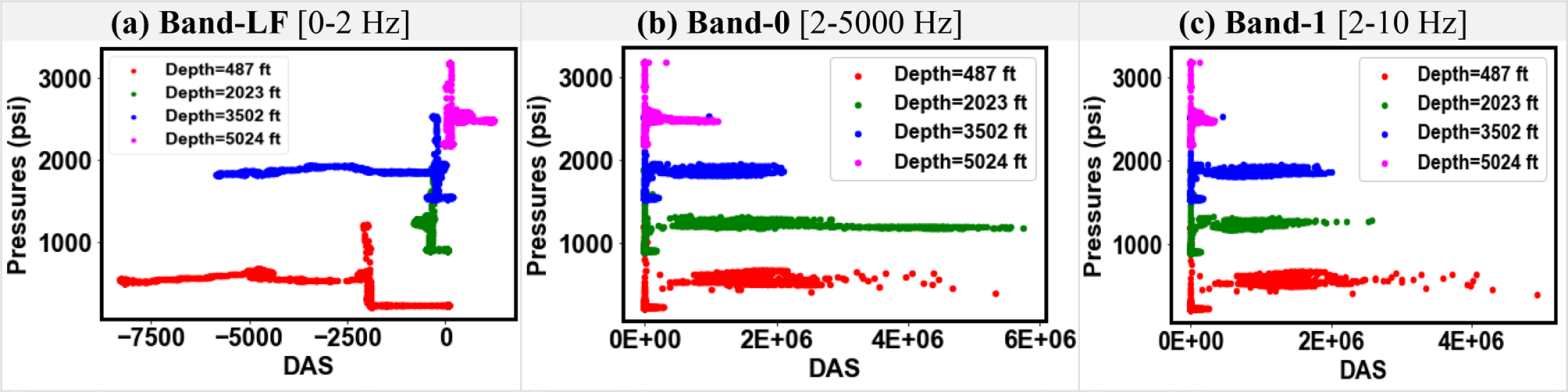
Cross-plots of pressure and different DAS frequency bands for Dataset-2.

Figures 9 and 10 show the cross-plots between DAS and DTS for the DAS frequency Bands-LF, 0, and 1 for Dataset-1 and Dataset-2, respectively. The high-frequency Bands 0 and 1 show a similar DAS-DTS relationship which is distinct from the low-frequency DAS data in Band-LF. For example, Fig. 9a shows a linear DAS-DTS relationship which is not seen for Figs. 9b and c. For the higher frequency bands in both datasets, the relationship between DTS and DAS cannot be clearly explained. Figures 11 and 12 show the cross-plots between DAS and pressure at various depths for Datasets-1 and Dataset-2, respectively. Again, the higher frequency DAS Bands 0 and 1 show a similar relationship with pressure, while the DAS Band-LF shows a more distinct trend. The exploratory data analysis demonstrates the unique properties of low-frequency DAS which can be attributed to the sensitivity of DAS to temperature and strain variations at low frequency^1, 23^.

### Single-depth predictive modeling

**Figure 13:**
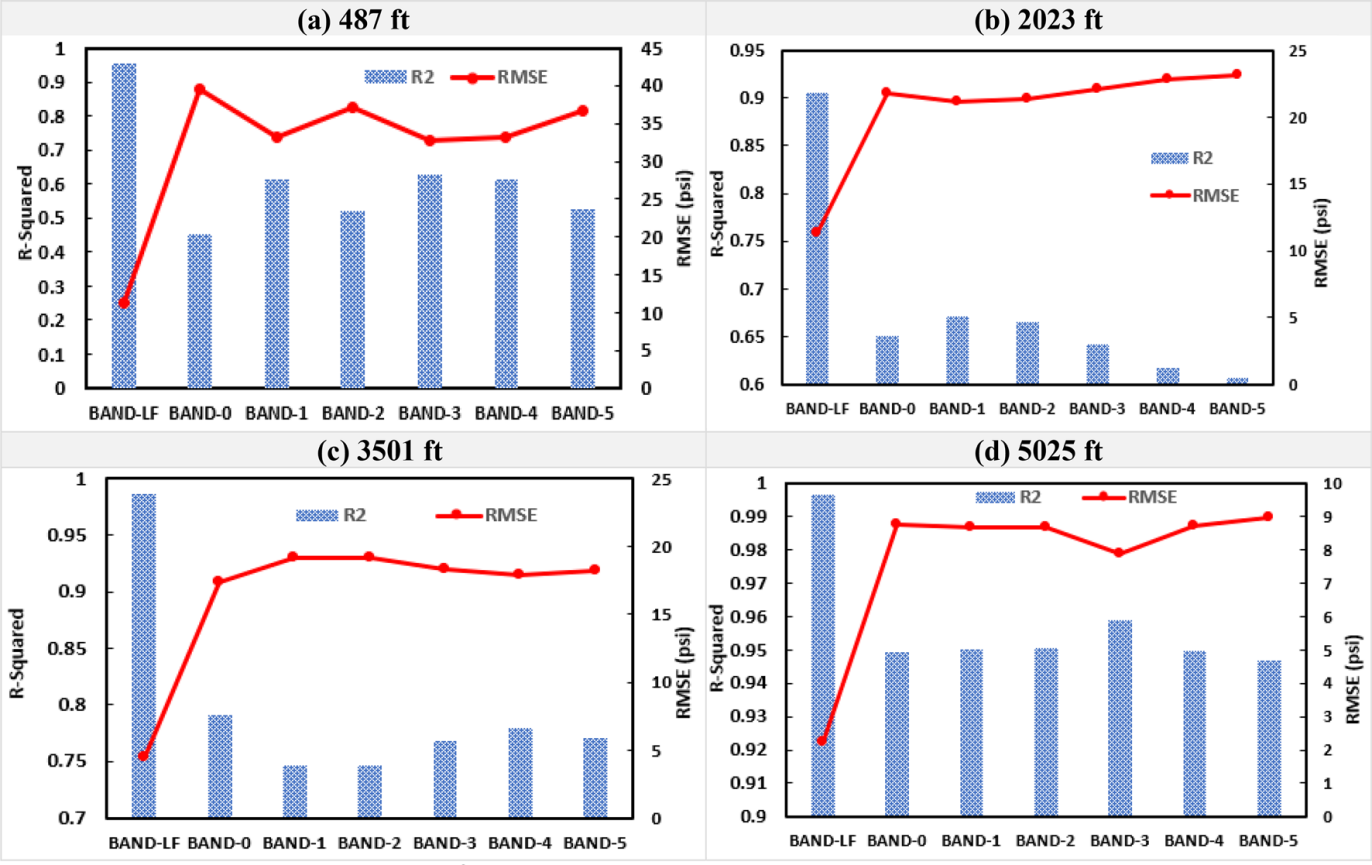
The RMSE and R^2^ values for the testing subsample at each gauge depth for Dataset-1.

**Figure 14:**
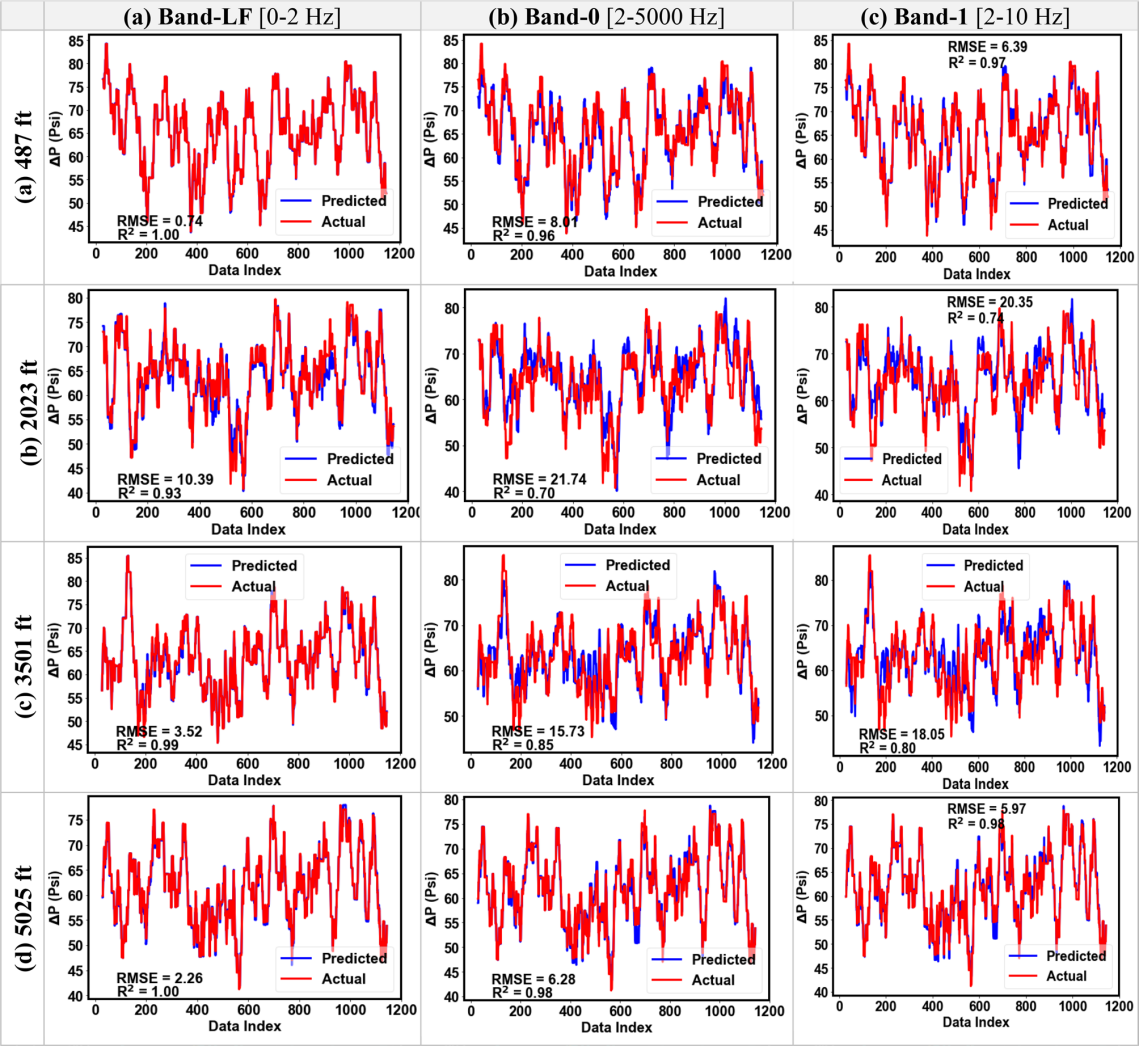
Pressure prediction using DAS Bands LF, 0, and 1 for the testing set for Dataset-1 (RMSE in psi).

In this section, the results of the single-depth analysis are discussed for both datasets at the four pressure gauge locations. The DAS and DTS were used as the input features while the change in pressure with respect to initial pressure at the first time-step (∆P) was used as the output variable (Table 3). Figure 13 shows the RMSE and R^2^ values for the pressure predictions of the testing sets of Dataset-1 at the four gauge locations for the seven DAS frequency bands. Figure 14 shows the predicted and the actual pressure profiles for the testing subsample for Dataset-1 for Bands-LF, 0, and 1. The predicted pressure profiles for Bands 2 to 5 for both the datasets are included in the supplementary material (Figs. S4, S5). The R^2^ of the Band-LF ranged from 0.90 to 0.99 with an average performance across all depths of 0.97, while the average R^2^ value across all depths for the higher frequency bands ranged from 0.81 to 0.83. Similarly, the RMSE values varied between 0.8 to 11.4 psi for the Band-LF and between 8 to 23 psi for the higher frequency bands. The average RMSE across all four depths was 4.7 psi for Band-LF compared to 14.2 psi average RMSE for the higher frequency bands. The results clearly demonstrate that the low-frequency DAS data gives a more accurate prediction for pressure for Dataset-1.

**Figure 15:**
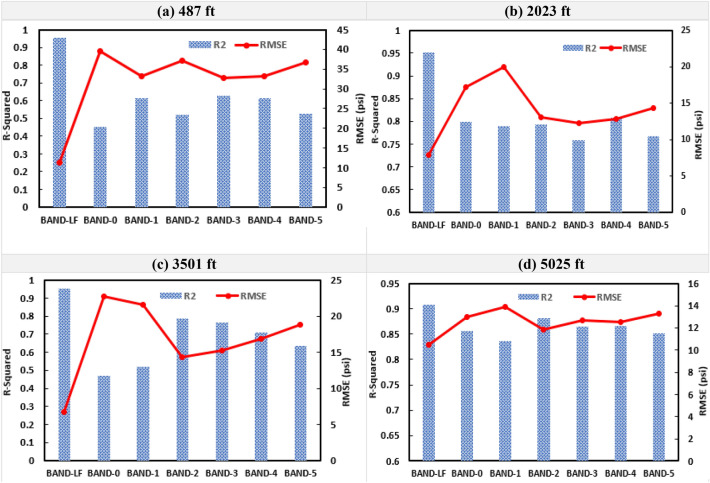
The RMSE and R^2^ values for the testing subsample at each gauge depth for Dataset-2.

**Figure 16:**
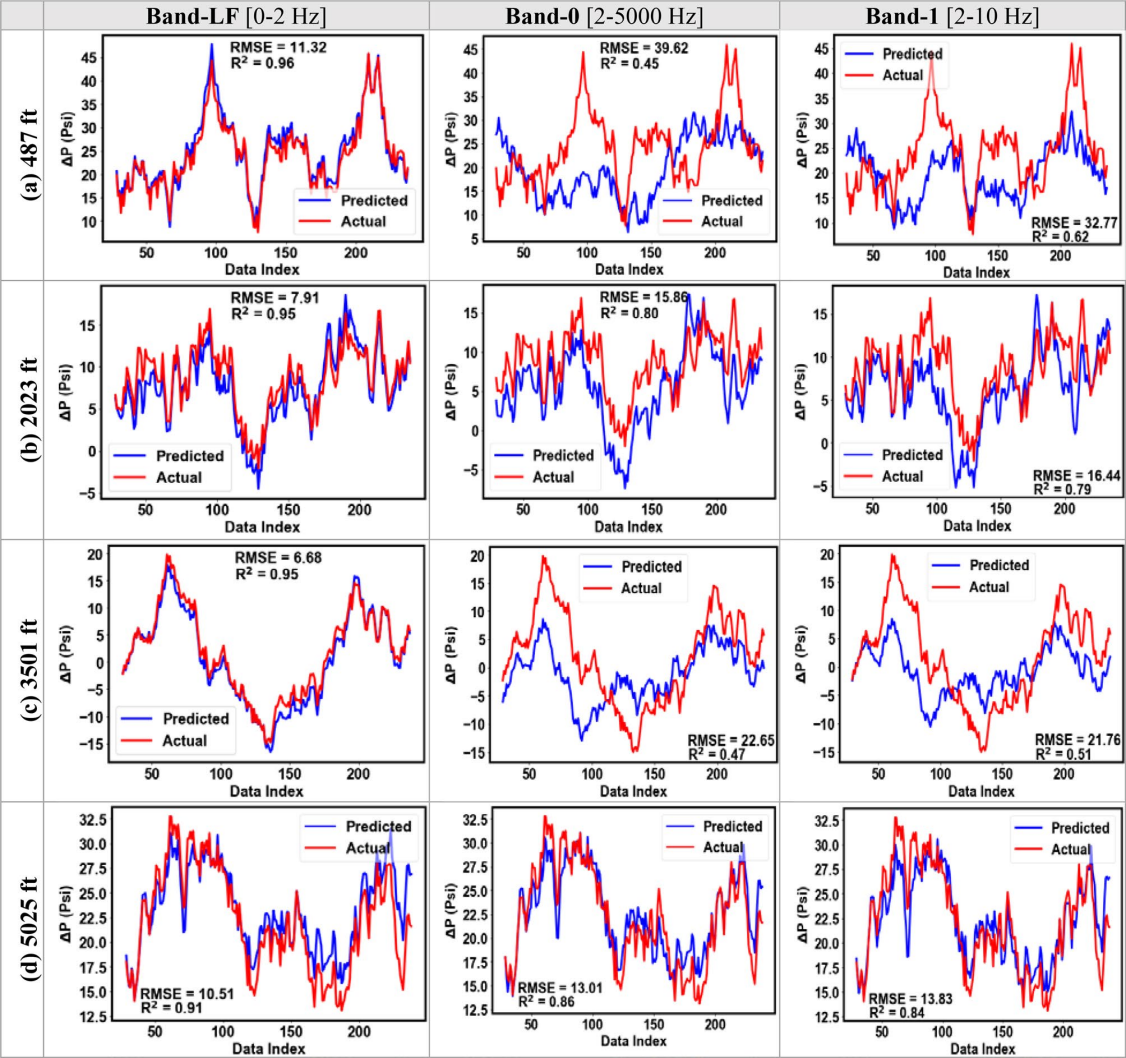
Pressure prediction using DAS Bands LF, 0, and 1 for the testing set for Dataset-2 (RMSE in psi).

Figure 15 shows the results from single-depth pressure prediction for Dataset-2 across the four gauge depths for the testing subsample. The R^2^ values for the Band-LF ranged from 0.90 to 0.96 with an average of 0.94 across all depths. While the average R^2^ values for the higher frequency DAS bands ranged from 0.64 to 0.75. Similarly, the RMSE values varied between 6.7 to 11.3 psi for the Band-LF and between 12.3 to 39.5 psi for the higher frequency bands. Figure 16 compares the predicted and the actual pressure trends for the testing subsample for Dataset-2. Similar to Dataset-1, Figs. 15 and 16 clearly demonstrate that the random forest model using low-frequency DAS as input gives a more accurate prediction of pressure.

### Distributed pressure predictive modeling

**Figure 17:**
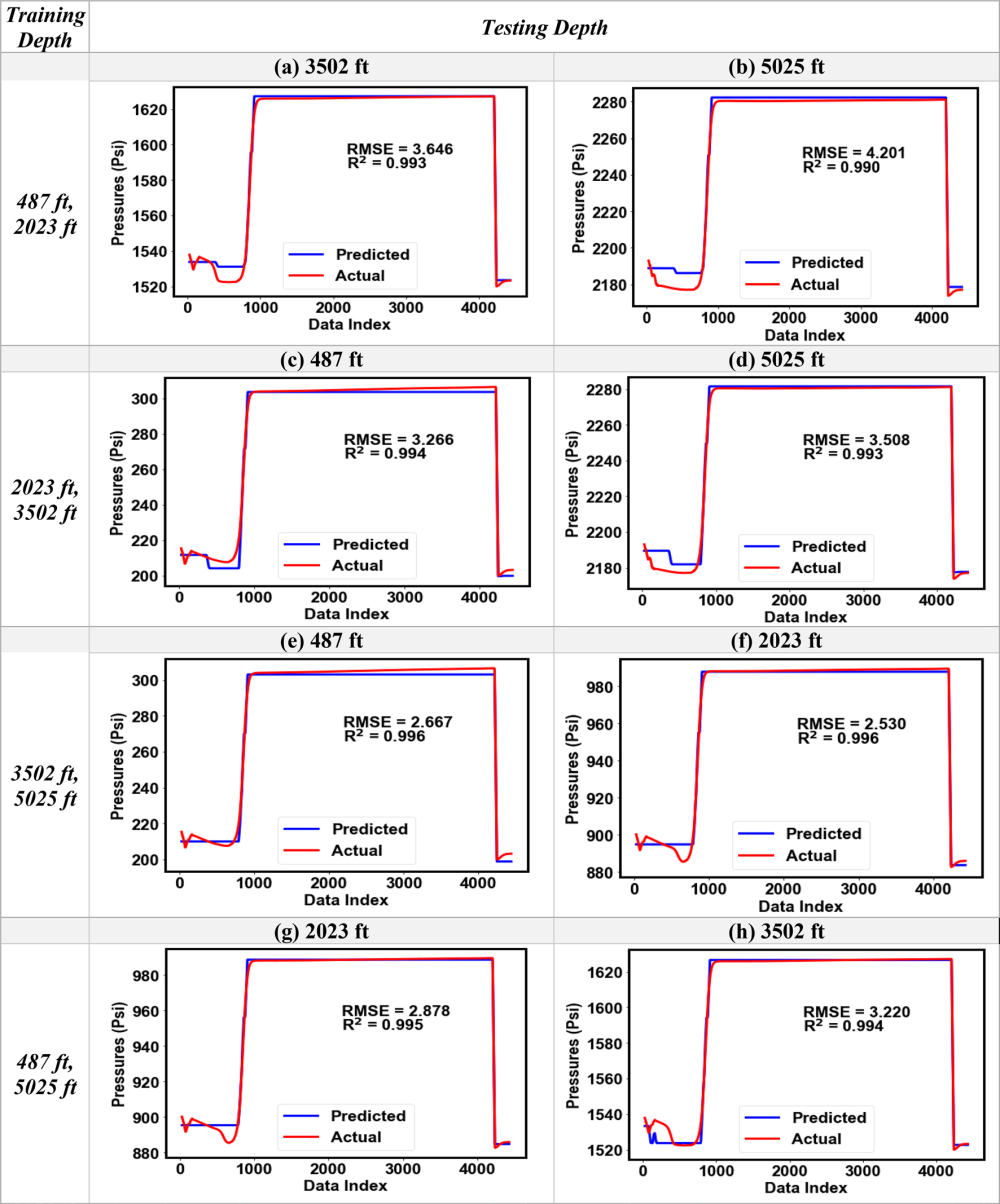
Comparison of the predicted and the actual pressure profiles obtained for the distributed pressure analysis for different combinations of training and testing depths for Dataset-1 (RMSE in psi).

**Figure 18:**
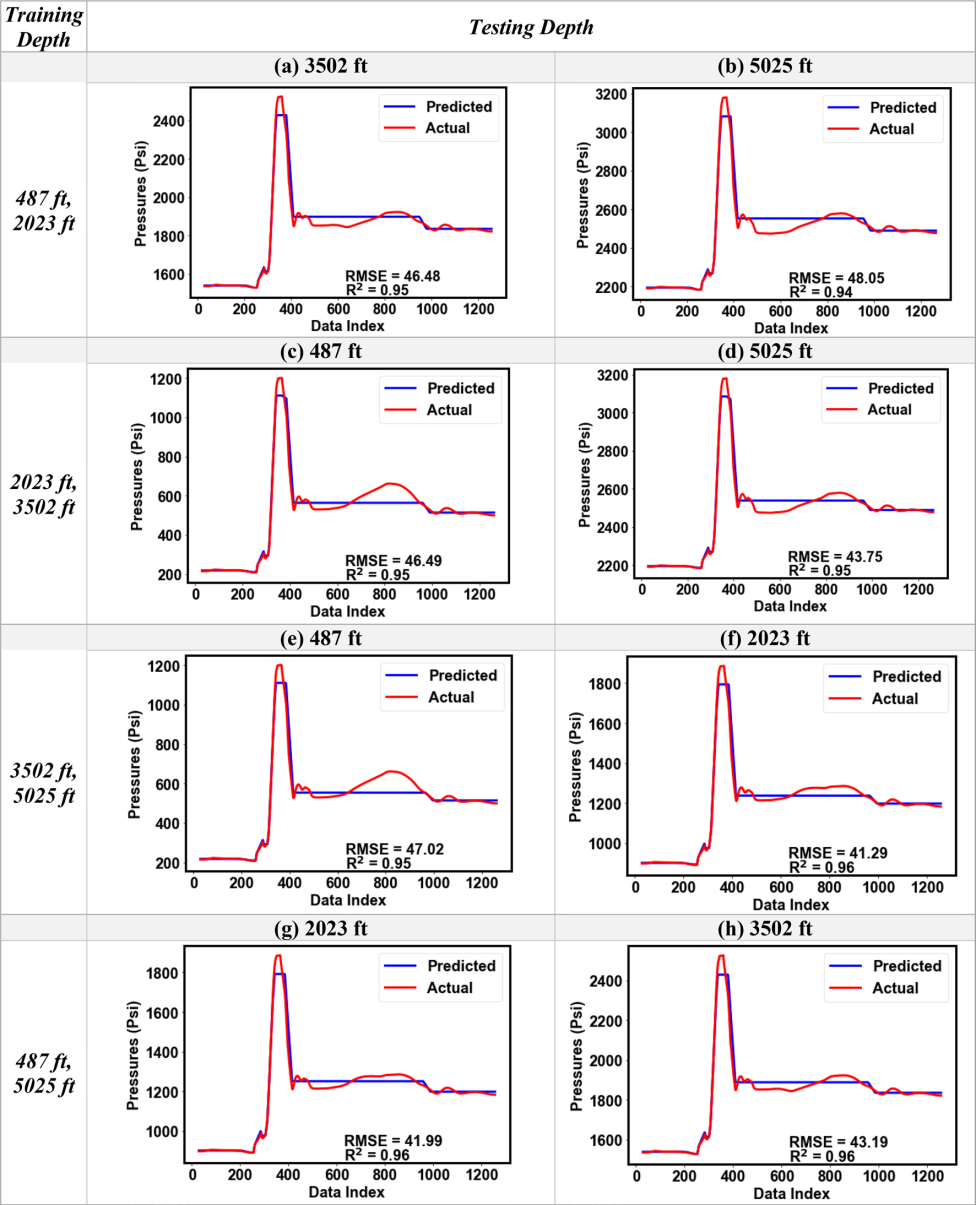
Comparison of the predicted and the actual pressure profiles obtained for the distributed pressure analysis for different combinations of training and testing depths for Dataset-2 (RMSE in psi).

The results from the single-depth pressure modeling clearly established that the low-frequency DAS (or Band-LF) gave a consistently better performance compared to the higher frequency DAS bands (> 2 Hz). Therefore, for the distributed pressure modeling, we used the input variables of DAS Band-LF, DTS, and elapsed time as the input features, and the change in pressure from the original (ΔP) as the output for the random forest model. Training of the model was performed with datasets from any two gauge depths while the resulting model was used to predict pressures at the other two depths. Figure 17a–h show the predicted versus the actual pressure plots for eight different scenarios, for Dataset-1 and Datset-2, respectively. For example, in Fig. 17a the DTS, DAS and pressure data at 487 ft and 2023 ft were used for training the random forest model and the trained model was used to predict pressures at 3502 ft using the DAS Band-LF and DTS at that depth. The R^2^ values for Dataset-1 for all eight scenarios were higher than 0.99 with the RMSE ranging between 2.5 to 4.2 psi. Similarly, Dataset-2 results in Fig. 18a–h show strong model performance with R^2^ greater than 0.95 in all cases. Although for Dataset-2 the RMSE was higher (44.7 psi) compared to Dataset-1, which is likely due to the dynamic effects resulting from water circulation.

## Discussion

Prediction of downhole pressure is crucial for wide-ranging potential applications including the management and evaluation of petroleum, geothermal, and groundwater resources. For oil operators, downhole pressure monitoring supports the determination of well productivity, estimation of flow rates, and sizing of surface and downhole equipment. The industry primarily relies on downhole and surface gauges to meet its pressure data needs, however, this often results in a deficiency of crucial data, due to the low spatial and temporal resolution achieved from gauges which only provide measurement at a handful of locations. Distributed pressure measurement simultaneously along the entire wellbore in real-time will give the operators and drillers never-before-seen visibility of the dynamics of fluid flow along the well and may reduce exposure to incidents and improve reservoir management.

Although the adoption of DAS and DTS is increasing rapidly, well-scale or field-scale distributed pressure sensing has not been reported using these measurements. This study presents the first well-scale application of fiber optics data for pressure prediction. To model the pattern in the data, we have trained a machine learning algorithm and then used the developed model to predict the pressure data at different depths. In a typical oilfield scenario, surface and downhole pressure gauges are commonly available which can be used for the model training, in conjunction with the DTS and DAS (which includes low-frequency data). The trained model can then be used for the distributed pressure prediction at various locations where DTS and DAS data is available. While data from two downhole gauge locations was used for training the model to demonstrate the workflow, even a single pressure gauge data may also be used for model training if that is the only pressure measurement available with co-located DAS and DTS information. 

The single-depth analysis showed that the low-frequency DAS (combined with DTS) consistently demonstrated superior capability to predict pressure compared to the higher frequency DAS (> 2 Hz). A plausible explanation for the better performance shown by the Band-LF (0–2 Hz) is the higher DAS sensitivity to temperature and strain in the low-frequency range. The pressure response to the fluid compression in turn is related to the longitudinal strain experienced by the fiber through the mechanical properties of the fiber (as discussed in Sec. 1.2). The results are consistent with those from some recent studies that have also shown that low-frequency DAS gives a better correlation with pressure. For instance, Becker et al.^1^ in their lab-scale experiment showed that the low-frequency DAS band (100 mHz) showed higher sensitivity to fluid pressure. In our well-scale experiments, the pressures investigated were up to 3200 psi whereas the maximum pressure in the Becker study was less than 1 psi. This study demonstrates that low-frequency DAS combined with DTS can be used for distributed pressure measurement at well-scale.

## Conclusions

This study presents the first well-scale application of distributed fiber-optic data for pressure prediction. The complex relationship between DAS, DTS, and pressures were modeled by training a machine-learning algorithm and the developed model was used to predict the pressure at different depths in a 5163 ft. deep weelbore. In a typical oilfield scenario, surface and downhole pressure gauges are commonly available, which can be used for model training, and subsequently the trained model can be used to predict pressure at different spatial locations where DAS and DTS information is available. The results demonstrate the frequency dependence of the pressure measured by the optical fiber. The low-frequency DAS components (< 2 Hz), together with DTS gave more accurate pressure predictions, as compared to the high-frequency DAS components. In the single-depth analysis with low-frequency DAS, the average coefficient of determination (or R^2^) was about 0.96 and the average RMSE was about 7 psi, for the two datasets analyzed. For the distributed pressure analysis the average R^2^ was over 0.95 and average RMSE was 24 psi for the two datasets for all depths analyzed, demonstrating strong model performance. This study presents a novel application of the low-frequency DAS combined with DTS for distributed pressure measurement at well-scale.

## Supplementary Information

Supplementary Information.
