# Supplementary material for: Well-scale demonstration of distributed pressure sensing using fiber-optic DAS and DTS
Source: Sci Rep. 2021 Jun 14;11:12505. doi: 10.1038/s41598-021-91916-7 (PMC8203739; doi:10.1038/s41598-021-91916-7)
Supplement: Supplementary file 1 — Supplementary Information. [file 41598_2021_91916_MOESM1_ESM.docx]

Figs. S1 and S2 show the DAS waterfall plots for the higher frequency Bands-3, 4, and 5 for Dataset-1 and Dataset-2, respectively. A detailed interpretation of the gas signature is included in the references provided in the main paper^23,24^.

**Figure S1:** Waterfall plots showing the DAS values in the different frequency bands for Dataset-1.

**Figure S2:** Waterfall plots showing the DAS values in the different frequency bands for Dataset-2.

The proposed pressure prediction workflow (Fig. 7) was implemented using different machine learning algorithms to select the most robust technique. All the algorithms were implemented using the Scikit-learn^1^, Tensorflow^2^ and XGBoost^3^ modules in python. The details of the RF, GBM^4,5^, XGBoost^3^, SVR^6,7^ and different architectures of shallow ANN^8,9^ that were compared in the study are summarized in Table S1.

| **Machine Learning Algorithm** | **Model Architecture** | **Model Hyperparameters** | **Running Time (s)** |
| --- | --- | --- | --- |
| **Random Forest (RF)** | - | Number_of_trees=100,learning_rate=0.1,min_samples_split=2,splitting_criteriob='mse' | 0.95 |
| **Gradient Boosting (XGB)** | - | Number_of_trees=100,learning_rate=0.1,min_samples_split=2,splitting_criteriob='mse' | 0.825 |
| **Extreme Gradient Boosting (GB)** | - | Number_of_trees=100,learning_rate=0.1,min_samples_split=2,splitting_criteriob='mse' | 0.9 |
| **Support Vector Regressions (SVR)** | - | kernel='rbf',gamma='scale',regularization_parameter (C) =1, epsilon=0.1 | 0.92 |
| **Artificial Neural Network**  **(ANN-1)** | 2:10:1 | optimizer='adam', loss='mse',batch_size=10, epochs=1000,activation function=ReLU | 244 |
| **Artificial Neural Network**  **(ANN-2)** | 2:10:10:1 | optimizer='adam', loss='mse',batch_size=10, epochs=1000,activation function=ReLU | 248 |
| **Artificial Neural Network**  **(ANN-3)** | 2:10:20:10:1 | optimizer='adam', loss='mse',batch_size=10, epochs=1000,activation function=ReLU | 266 |

**Table S1:** Machine learning models compared in this study.

Fig. S3 show the R^2^ and RMSE scores for the different machine learning algorithms. The values shown are the mean values across the four gauge depths using the DAS Band-LF (0 to 2 Hz) for Dataset-2. The Random Forest model used in the main body of work consistently showed similar or better performance (i.e. high R^2^ and low RMSE) compared to the other techniques investigated in all seven frequency bands analyzed in both the datasets. The ANN architecture when deep enough may also give improved results but the computational time inhibits its usage.


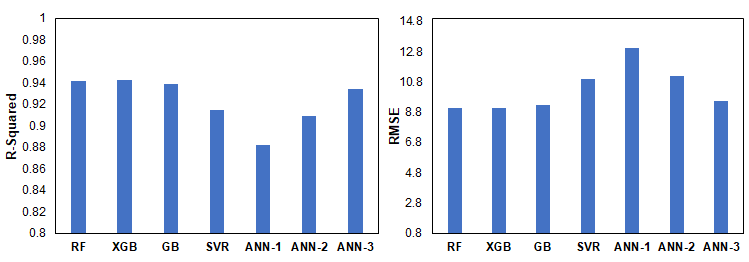


**Figure S3**: R^2^ and RMSE of the predictions obtained using the different machine learning algorithms for Dataset-2.

The pressure prediction results for the single-depth analysis for Bands 2 to 5 for the testing sets are presented in Figs. S4 and S5 for Dataset-1 and Dataset-2, respectively. The results support the conclusion in the main manuscript that the low-frequency band gives better performance for pressure prediction (i.e. highest R^2^ and low RMSE) as compared to the higher frequency bands.


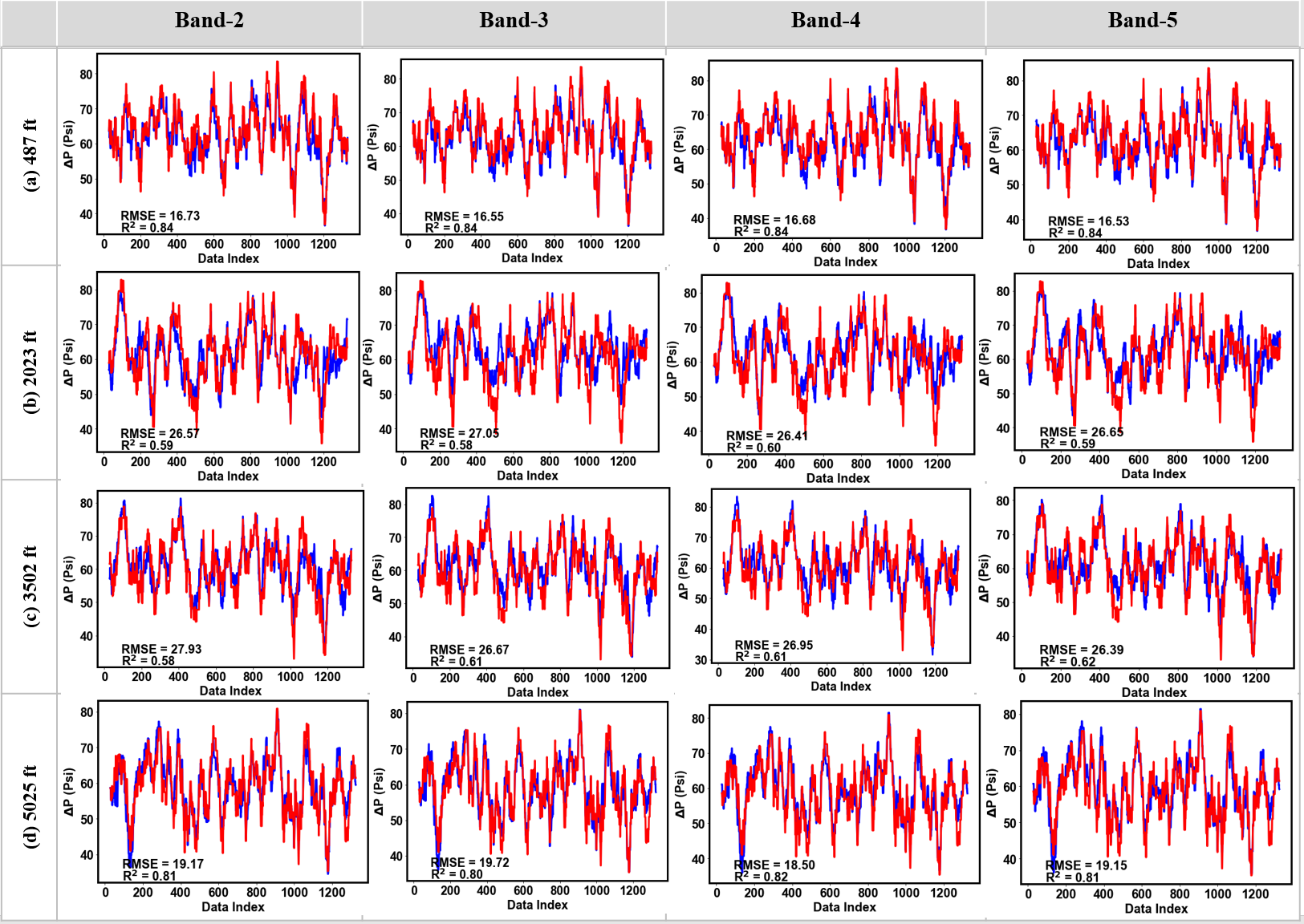


**Figure S4:** The series plots of pressures predicted from DAS Bands 3, 4, and 5 for Dataset-1 (RMSE in psi).


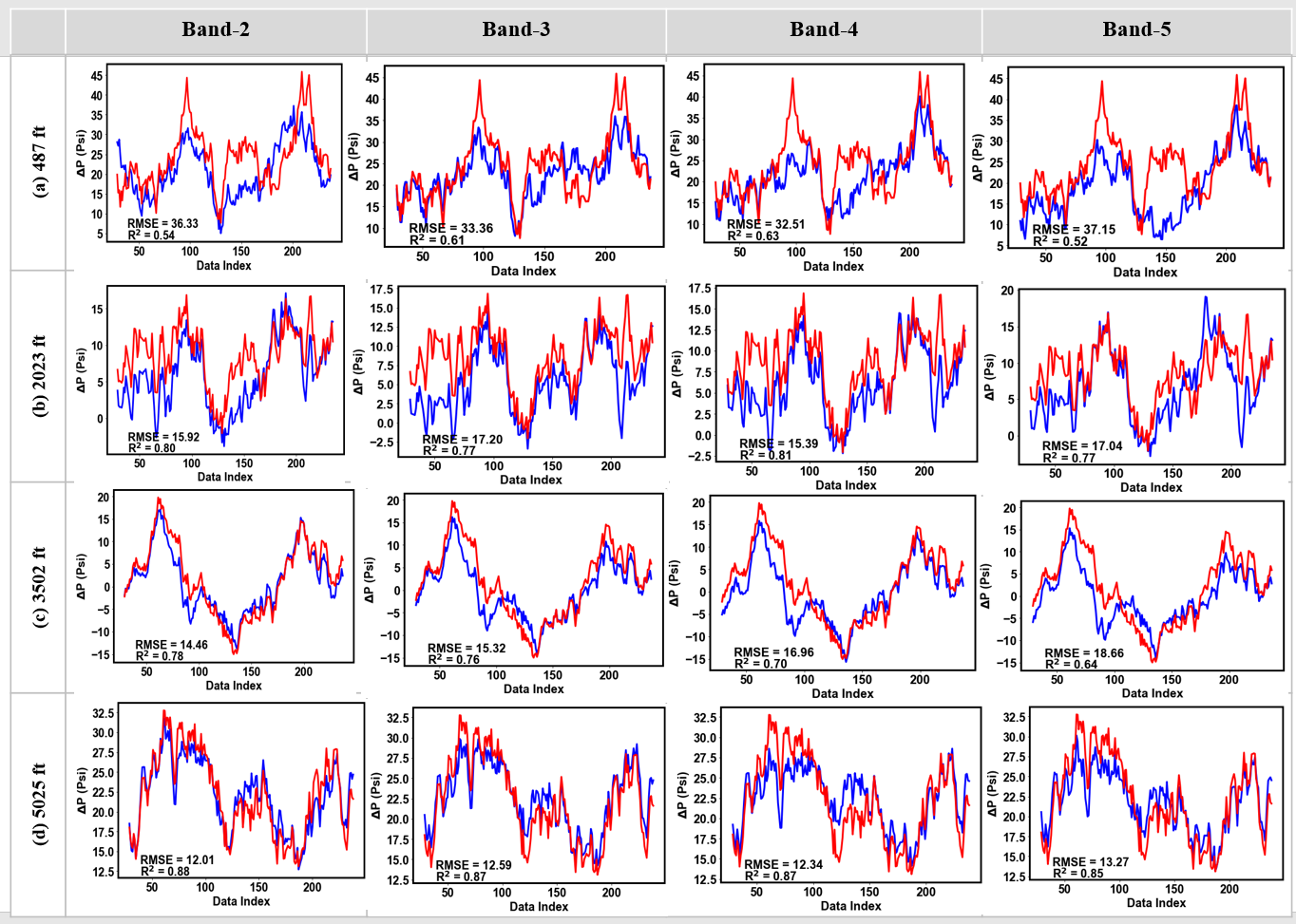


**Figure S5:** The series plots of pressures predicted from DAS Bands 3, 4, and 5 for Dataset-2 (RMSE in psi).

**Reference**

1. Pedregosa, F. *et al.* Scikit-learn: Machine Learning in Python. (2012).

2. Abadi, M. *et al.* TensorFlow: Large-Scale Machine Learning on Heterogeneous Distributed Systems. (2016).

3. Chen, T. & Guestrin, C. XGBoost. in *Proceedings of the 22nd ACM SIGKDD International Conference on Knowledge Discovery and Data Mining* 785–794 (ACM, 2016). doi:10.1145/2939672.2939785

4. Friedman, J. H. machine. *Ann. Stat.* **29**, 1189–1232 (2001).

5. Friedman, J. H. Stochastic gradient boosting. *Comput. Stat. Data Anal.* **38**, 367–378 (2002).

6. Cortes, C. & Vapnik, V. Support-vector networks. *Mach. Learn.* **20**, 273–297 (1995).

7. Vapnik, V. *The Nature of Statistical Learning Theory*. (Springer, 1995).

8. McCulloch, W. S. & Pitts, W. A logical calculus of the ideas immanent in nervous activity. *Bull. Math. Biophys.* **5**, 115–133 (1943).

9. Schmidhuber, J. Deep learning in neural networks: An overview. *Neural Networks* **61**, 85–117 (2015).
